# Differential impact of two doses of antithymocyte globulin conditioning on lymphocyte recovery upon haploidentical hematopoietic stem cell transplantation

**DOI:** 10.1186/s12967-015-0748-x

**Published:** 2015-12-30

**Authors:** Jiangying Liu, Lan-Ping Xu, Zhilei Bian, Ying-Jun Chang, Yu Wang, Xiao-Hui Zhang, Xiao-Jun Huang

**Affiliations:** Peking University People’s Hospital, Peking University Institute of Hematology, Beijing Key laboratory of Hematopoietic Stem Cell Transplantation, 11 Xizhimen South Street, Beijing, 100044 China

**Keywords:** Hematopoietic stem cell transplantation, Antithymocyte globulin (ATG), Immune reconstitution, Viral infection, Epstein-Barr virus (EBV)

## Abstract

**Background:**

In vivo depletion of host T cells with antithymocyte globulin (ATG) is a common strategy for preventing graft-versus-host disease in allogeneic hematopoietic stem cell transplantation (HSCT). The total dose of ATG in conditioning regimens appears to be an important factor that influences the outcome in recipients of transplants. However, the optimal ATG dosage has not been established to date. It remains unclear whether, in the setting of haploidentical HSCT (haploHSCT), different doses of ATG might exert differential influences on the recovery of lymphocyte subpopulations.

**Methods:**

This retrospective study analyzed lymphocyte recovery and its correlation to viral infection in two groups of patients that received different doses of ATG before haploHSCT. We performed flowcytometry to determine immunophenotypes of CD19^+^ B cells and CD3^+^, CD4^+^, CD8^+^, CD4^+^CD45RA^+^, CD4^+^CD45RO^+^, CD4^+^CD28^+^, CD8^+^CD28^+^, and CD4^−^CD8^−^ T cells.

**Results:**

We found that, compared to 6 mg/kg, 10 mg/kg ATG significantly hampered the recoveries of CD4^+^, CD4^+^CD45RA^+^, and CD4^+^CD45RO^+^ T cells in the first 2 months following haploHSCT. Similarly, compared to 6 mg/kg, the 10 mg/kg dose of ATG negatively influenced the recoveries of CD4^−^CD8^−^ and CD8^+^CD28^+^ T cells; recovery was delayed for 6 and 12 months after transplantation, respectively. Moreover, we showed that an increase in Epstein-Barr virus (EBV) infections, associated with the higher dose of ATG, was correlated with the delayed recovery of CD4^−^CD8^−^ double negative T cells.

**Conclusions:**

The present study revealed a differential impact of different ATG conditioning doses on the recoveries of T cell subpopulations post-haploHSCT. This study was the first to connect the recovery of CD4^−^CD8^−^ T cells to the risk of EBV infection after HSCT. These findings will facilitate optimization of the ATG conditioning dosage and improve the outcome of patients with leukemia that receive haploHSCT.

## Background

Haploidentical hematopoietic stem cell transplantation (haploHSCT) constitutes an important alternative for patients with leukemia that lack a donor with matching human leukocyte antigen (HLA). However, graft-versus-host disease (GVHD) is a frequent, life-threatening complication of haploHSCT. To prevent GVHD, patients often receive antithymocyte globulin (ATG), a polyclonal immunoglobulin preparation obtained by immunizing rabbits or horses with human thymocytes or T cell lines. This strategy of in vivo T cell depletion with ATG has been quite successful in preventing the development of both acute and chronic GVHD after hematopoietic stem cell transplantation (HSCT) from HLA-matched, unrelated donors [1–3]. Studies from our group and others have suggested that ATG can also overcome the negative influence of HLA-mismatched HSCT [4–7].

However, the virtual depletion of T cells increases both the risk of developing a severe infection and the risk of a malignancy relapse after HSCT. Therefore, it has become critical to determine a suitable dose of ATG in conditioning regimens [8]. Although the total dose of ATG appears to have an important influence on the overall clinical outcome of transplantation, studies on the impact of different ATG doses have produced inconsistence [9–11], and an optimal dose has not been established.

It has been recognized that viral infections and other complications of HSCT are closely associated with the reconstitution of the immune system. A growing number of studies have demonstrated the negative impact of ATG on the recovery of lymphocytes after transplantation [12–14]. In the haploHSCT setting, our previous prospective study reported that, compared to 6 mg/kg ATG, 10 mg/kg ATG conditioning regimen increased the risk of EBV infection, but reduced the incidence of grades III–IV acute GVHD [15]. Although patients that received higher ATG dose showed delayed recoveries of CD19^+^ B cells, CD3^+^ T cells, and CD4^+^ T cells 30 days after transplantation, it was not clear whether lymphocyte recovery at later stages and the recoveries of differential T cell subpopulations were affected by different doses of ATG. Moreover, the cause-consequence relationship between immunodeficiency and the occurrence of viral infections was not demonstrated in this context.

In the present study, we extended the comparison between the two different ATG-conditioning doses in the context of haploHSCT to include more lymphocyte subpopulations and longer follow-up times. Thus, in addition to CD19^+^ B cells and CD3^+^ and CD4^+^ T cells, we studied recoveries of CD8^+^, CD4^+^CD45RA^+^, CD4^+^CD45RO^+^, CD4^+^CD28^+^, CD8^+^CD28^+^, and CD4^−^CD8^−^ T cells for an extended time course (12 months after transplantation). Our results showed hampered recovery of some T cell subsets and a correlation between the delayed recovery and increased risk of EBV infection with 10 mg/kg ATG treatment. These results emphasized the need to optimize the ATG dosage in conditioning regimens to facilitate recovery of the immune system and improve the outcome for recipients of haploHSCT.

## Methods

### Patients and study design

The aim of this study was to compare lymphocyte recovery and its correlation to viral infections between patients that received two different doses of ATG as conditioning for haploHSCT. We retrospectively screened participants from an open-label, prospective, randomized trial (No. ChiCTR-TRC-11001761), which included 224 patients randomly assigned to receive either 6 mg/kg or 10 mg/kg ATG [15]. All subjects were diagnosed with standard-risk hematological malignancies and underwent haploHSCT in our institute from December 2010 to May 2012. The Ethics Committee of Peking University Institute of Hematology approved the study protocol. All recipients and their donors signed consent forms. Because immune reconstitution was neither the primary nor the secondary end point in that prospective study, only 60 subjects had been monitored for lymphocyte recovery at serial time points (30, 60, 90, 180, and 360 days post-transplantation). Therefore, these 60 subjects were recruited for the current study.

Patients were conditioned with intensive chemotherapy-based regimens (mainly busulfan, cyclophosphamide, and ATG), as described in detail previously [15]. ATG (thymoglobulin; rabbit ATG, Imtix Sangstat, Lyon, France) doses were administered from day −5 to day −2 before transplantation. Patients that received 6 mg/kg ATG (ATG-6 group) were administrated 1.5 mg/kg per day; patients that received 10 mg/kg ATG (ATG-10 group) were administrated 2.5 mg/kg per day. The transplant procedures, GVHD prophylaxis (including cyclosporine A and short-term methotrexate with mycophenolate mofetil) and treatment, and evaluations of acute GVHD and viral infections/reactivations were described previously [15].

### Immunophenotyping analysis

Immunophenotyping for B and T lymphocytes was performed with flowcytometry post-haploHSCT. Briefly, fresh peripheral blood cells were stained with fluorochrome-labeled monoclonal antibodies against cluster of differentiation (CD) cell surface molecules, CD19, CD3, CD4, CD8, CD45RO, CD45RA, and CD28 (Beckton Dickinson, San Jose, CA, USA). Polychromatic flow cytometric analyses were performed with a custom 7-laser high resolution LSRII cell analyzer (Beckton Dickinson).

### Statistical analysis

Statistical differences between groups in age, incidences of acute GVHD and viral infections, and recoveries of lymphocyte subpopulations at different time points were analyzed with the Mann–Whitney U test. Statistical differences between the two groups in gender and type of primary disease were analyzed with the Chi squared test. Bivariate correlations between the recoveries of T cell subpopulations and the incidence of EBV infection were analyzed with the Spearman test. Statistical significance was defined as *P* ≤0.05, based on a two-tailed test. All calculations were carried out with SPSS 16.0 statistical software (SPSS Inc, Chicago, IL, USA).

## Results

### Characteristics of patients

The characteristics of patients in the two different ATG groups are summarized in Table 1. Previous studies suggested that the age of recipients and the numbers of CD34^+^ cells in grafts could influence immune recovery. In the present study, there were no statistical differences in age or the numbers of CD34^+^ cells in grafts between groups. Moreover, the gender ratio and types of primary disease were similar between the ATG-6 and ATG-10 groups (Table 1). In addition, all included subjects underwent the same procedures of donor priming (recombinant human granulocyte-colony stimulating factor), graft harvesting (peripheral blood progenitor cells and unmanipulated bone marrow), and GVHD prophylaxis (cyclosporine A and short-term methotrexate with mycophenolate mofetil).

**Table 1:** Clinical characteristic of patients

	ATG-6	ATG-10	*P* value
Subjects, n	29	31	
Age, years, median (range)	22 (16–48)	29 (15–48)	0.11
Gender, n (%)			
Male	18 (62.1)	24 (77.4)	0.19
Female	11 (37.9)	7 (22.6)	
Diagnosis, n (%)			
AML	12 (41.4)	14 (45.2)	0.77
ALL	12 (41.4)	12 (38.7)	
CML	2 (6.9)	3 (9.6)	
MDS (RAEB)	3 (10.3)	2 (6.5)	
CD34^+^ cells in graft, ×10^6^/kg, median (range)	2.4 (0.7–4.9)	3.1 (0.3–7.8)	0.32
Donor type	Related donors		
HLA typing	1–3/6 Mismatch		
Stem cell source	BM + PB		
Conditioning regimen	BU + CY + ATG		
GVHD prophylaxis	CSA + MMF + MTX		

*ATG* antithymocyte globulin, *AML* acute myeloid leukemia, *ALL* acute lymphoblastic leukemia, *BM* bone marrow, *BU* busulfan, *CML* chronic myeloid leukemia, *CSA* cyclosporine A, *CY* cyclophosphamide, *HSCT* hematopoietic stem cell transplantation, *MDS* myelodysplastic syndrome, *MMF* mycophenolate mofetil, *MTX* methotrexate, *PB* peripheral blood

### Recoveries of T lymphocyte subpopulations were delayed with 10 mg/kg ATG

We first analyzed the peripheral white blood cell (WBC) and the absolute lymphocyte counts determined in routine blood tests from day 30 to 360 after haploHSCT. As shown in Table 2, the median levels of total WBC and lymphocyte counts were not significantly different between the two ATG groups at the indicated time points post-transplantation. This finding indicated that the recoveries of peripheral WBCs and lymphocytes were not affected by the dose of ATG in the conditioning regimen.

**Table 2:** Peripheral white blood cells (WBC) and lymphocyte counts after ATG-conditioned haploHSCT, 10^9^/L

	Group	WBC	Lymphocyte
Day 30	ATG-6	5.9 (1.6–15.7)	0.3 (0.1–2.7)
	ATG-10	4.7 (2.0–18.1)	0.3 (0.1–2.3)
	*p* value	0.95	0.47
Day 60	ATG-6	4.9 (1.9–14.9)	1.1 (0.3–3.3)
	ATG-10	4.3 (0.1–26.1)	0.9 (0.0–10.8)
	*p* value	0.46	0.57
Day 90	ATG-6	4.5 (1.7–11.2)	1.6 (0.2–3.2)
	ATG-10	3.8 (1.0–9.5)	1.2 (0.2–6.2)
	*p* value	0.27	0.34
Day 180	ATG-6	5.2 (2.8–8.7)	2.0 (0.9–3.9)
	ATG-10	4.9 (0.5–7.2)	1.8 (0.2–4.8)
	*p* value	0.54	0.36
Day 360	ATG-6	5.8 (3.8–15.0)	2.4 (0.4–5.2)
	ATG-10	5.4 (2.0–9.6)	2.2 (0.9–4.7)
	*p* value	0.58	0.88

The recoveries of peripheral WBC and lymphocytes are indicated by the median (minimum, maximum) absolute cell counts

Next, we compared the recoveries of B and T lymphocytes between the ATG-6 and ATG-10 groups at serial time points and within 1 year after haploHSCT. We determined the absolute numbers of nine lymphocyte subpopulations with immunophenotyping, namely: CD19^+^ B cells, and CD3^+^, CD4^+^, CD8^+^, CD4^+^CD45RA^+^, CD4^+^CD45RO^+^, CD4^+^CD28^+^, CD8^+^CD28^+^, and CD4^−^CD8^−^ T cells (Table 3). On day 30 after HSCT, the median counts of nearly all lymphocyte subsets, except CD8^+^ T cells, were significantly lower in the ATG-10 group than in the ATG-6 group. That is, the difference between groups were statistically significant (*P* ≤ 0.05) in comparing median counts of CD4^+^, CD4^+^CD45RA^+^, CD4^+^CD45RO^+^, CD4^+^CD28^+^, CD8^+^CD28^+^, and CD4^−^CD8^−^ T cells by the end of the first month post-transplantation; however, differences in the numbers of CD19^+^ B cells and CD3^+^ T cells were only close to the significance (*P* = 0.07, Table 3). These results demonstrated that 10 mg/kg of ATG administration, in contrast to 6 mg/kg of ATG treatment, was associated with a delayed recovery of lymphocyte subpopulations as early as the first month following haploHSCT.

**Table 3:** Recoveries of lymphocyte subpopulations after ATG-conditioned haploHSCT, cells/μL

	Group	CD19^+^	CD3^+^	CD4^+^	CD8^+^	CD4^+^CD45RA^+^	CD4^+^CD45RO^+^	CD4^+^CD28^+^	CD8^+^CD28^+^	CD4^−^CD8^−^
Day 30	ATG-6	10.7 (0.3–28.1)	195.9 (7.8–1488)	47.6 (0.9–246)	104.8 (5.2–1273)	43.7 (0.6–207)	0.4 (0.0–3.1)	57.5 (0.7–1091)	48.3 (1.1–227)	21.9 (1.6–81.6)
	ATG-10	5.9 (0.4–406)	149.5 (2.1–1433)	20.1 (0.2–272)	82.7 (0.1–1283)	20.0 (0.0–268)	0.2 (0.0–2.3)	26.6 (0.1–736)	19.9 (0.1–260)	10.9 (0.9–92.7)
	p value	0.07	0.07	0.02	0.10	0.01	0.03	0.02	0.01	0.03
Day 60	ATG-6	15.9 (1.2–140)	940.8 (60.3–3406)	140.3 (20.2–1051)	722.0 (53.0–2666)	125.8 (17.8–763)	1.6 (0.1–19.0)	151.7 (0.4–922)	111.3 (1.1–481)	51.5 (1.6–369)
	ATG-10	13.1 (0.3–3778)	691.6 (24.8–6765)	81.2 (1.8–813)	533.9 (20.7–5262)	78.2 (1.6–704)	0.6 (0.0–94.7)	159.6 (4.1–2580)	72.9 (1.7–892)	22.1 (0.3–703)
	p value	0.54	0.53	0.04	0.71	0.03	0.04	0.39	0.05	0.02
Day 90	ATG-6	15.2 (2.8–102)	1127.2 (146–2859)	187.965 (27.2–810)	784.2 (62.6–2505)	159.6 (2.3–521)	1.2 (0.0–19.8)	195.8 (14.1–732)	106.9 (36.8–357)	64.7 (8.0–313)
	ATG-10	9.1 (0.3–356)	885.6 (0.2–5688)	113.4 (15.7–1162)	697.3 (0.2–4404)	109.4 (14.4–911)	0.7 (0.1–24.2)	116.7 (0.0–1054)	62.5 (0.0–496)	33.5 (0.0–182)
	p value	0.27	0.26	0.09	0.78	0.14	0.23	0.29	0.02	0.04
Day 180	ATG-6	62.0 (2.5–384)	1423 (333–3543)	256.2 (67.1–951)	1092 (171–2995)	206.5 (58.2–675)	2.6 (0.8–58.8)	185.9 (71.6–521)	224.5 (62.1–362)	83.9 (23.1–1284)
	ATG-10	61.6 (0.4–322)	1374 (106–4317)	244.5 (24.5–707)	1091 (75.9–3714)	210.0 (22.2–614)	3.0 (0.1–14.5)	161.1 (0.3–727)	131.8 (2.8–343)	39.8 (5.4–195)
	p value	0.68	0.39	0.49	0.99	0.73	0.68	0.32	0.06	0.01
Day 360	ATG-6	204.5 (23.0–689)	1688 (803–4680)	355.9 (178–1061)	1170 (367.7–4007)	271.8 (147–512)	4.6 (0.8–11.7)	229.3 (96.8–463)	294.9 (146–1134)	80.4 (10.0–883)
	ATG-10	82.7 (6.2–787)	1620 (625–4994)	261.3 (55.1–1579)	1018 (425–3308)	224.1 (54.4–1449)	4.8 (0.3–56.7)	173.8 (48.8–459)	200.3 (46.8–571)	78.2 (8.6–1297)
	p value	0.23	0.83	0.23	0.92	1.00	0.34	0.18	0.02	0.98

The median levels of CD4^+^, CD4^+^CD45RA^+^ and CD4^+^CD45RO^+^ T cells (representing naive and memory CD4^+^ T cells) in the ATG-10 group continuously declined over 60 days compared to levels in the ATG-6 group. After that, from 90 to 360 days after transplantation, the median counts of these three T-cell subsets were comparable between the two groups (Table 3). This finding suggested that the recoveries of total, naive, and memory CD4^+^ T cells were hamperAs presented ined by the higher dose of ATG in the early stage, but not the late stage, post-haploHSCT.

In contrast, the median counts of CD8^+^CD28^+^ T cells (representing normal cytotoxic T lymphocytes) were apparently lower at all time points in the ATG-10 group than in the ATG-6 group. The *P* value for comparisons at 30, 60, 90, and 360 days were always ≤0.05; nevertheless *P* value rose to 0.06 at 180 days (Table 3). This observation implied that the recovery of active CD8^+^ T cells was slowed by a higher dose of ATG for relatively long times, and this in turn, might have contributed to the outcomes of haploHSCT observed in this group.

Notably, the recovery of a special T-cell subpopulation defined as CD4^−^CD8^−^ T cells was also hampered in the ATG-10 group at 30, 60, 90, and 180 days, with *P* values <0.05 compared to the ATG-6 group. However, the median counts of CD4^−^CD8^−^ T cells were comparable between the two groups at 360 days after haploHSCT (Table 3). It was recognized that CD4^−^CD8^−^ T cells represent a small subpopulation of the normal immune system. Therefore, the impact of impairing the recovery of this double-negative T-cell subset during the first half year after ATG-conditioned haploHSCT requires further investigation.

### High dose ATG conditioning was associated with a lower incidence of acute GVHD but increased EBV reactivation after haploHSCT

Next, we evaluated whether different doses of ATG administration pre-transplantation might impact the incidence of acute GVHD (aGVHD). As shown in Table 4, the total incidence of aGVHD was significantly decreased in the ATG-10 group compared to the ATG-6 group (45.2 vs 72.4 %, *P* = 0.03), but there was no difference in the occurrence of significant and severe aGVHD (grades II–IV) between groups. The onset time of aGVHD was comparable between groups.

**Table 4:** Incidences of acute GVHD and CMV/EBV infections after ATG-conditioned haploHSCT

	ATG-6	ATG-10	*p* value
Acute GVHD, n (%)			
Total	21 (72.4)	14 (45.2)	0.03
Grade II–IV	11 (37.9)	8 (25.8)	0.32
Grade III–IV	4 (13.8)	2 (6.5)	0.35
Median day of onset grade II-IV aGVHD (range)	29 (10–42)	30 (9–80)	0.32
CMV reactivation, n (%)	22 (75.9)	24 (77.4)	0.89
Median day of onset CMV reactivation (range)	36 (22–77)	37.5 (22–102)	0.73
EBV reactivation, n (%)	2 (6.9)	10 (32.2)	0.02
Median day of onset EBV reactivation (range)	60.5 (47–74)	51 (22–102)	0.61

It was previously shown that a higher risk of viral infections was associated with the use of ATG. In the current study, the risk of cytomegalovirus (CMV) infection was similar in the ATG-10 and ATG-6 groups. However, Epstein–Barr virus (EBV) reactivation occurred more frequently in ATG-10 group than in the ATG-6 group (32.2 vs 6.9 %, *P* = 0.02). The onset times of CMV and EBV reactivations were not statistically different between the two ATG-dose groups (Table 4).

### Recovery of CD4^−^CD8^−^ T cells was negatively correlated with EBV reactivation after 10 mg/kg ATG conditioning

Given the observation that a higher ATG conditioning dose influenced both the recovery of T lymphocyte subpopulations and the incidence of EBV reactivation in the current study, we were interested in determining whether these two phenotypes were correlated. Accordingly, we performed a Bivariate Correlations analysis between the median counts of T cell subpopulations that showed significantly delayed recoveries in the ATG-10 group (Table 3) and the incidence of EBV reactivation in that group. Among five T lymphocyte subsets (CD4^+^, CD4^+^CD45RA^+^, CD4^+^CD45RO^+^, CD8^+^CD28^+^, and CD4^−^CD8^−^ T cells), only the recovery of CD4^−^CD8^−^ T cells on day 30 was significantly correlated to the occurrence of EBV reactivation (Spearman’s r_p_ = −0.378, *P* = 0.036, Table 5). The correlation between the recovery of CD4^−^CD8^−^ T cells on day 90 and EBV infection nearly reached statistical significance (Spearman’s r_p_ = −0.352, *P* = 0.066, Table 5). We note that median onset time of EBV reactivation was 51 days (range 22–102 days) after transplantation in the ATG-10 group (Table 4). Based on this observation, we speculated that, among patients that received a higher dose of ATG, the delayed recovery of CD4^−^CD8^−^ T cells in the first month increased the risk of EBV infection. In turn, an EBV infection might slow down the recovery of CD4^−^CD8^−^ T cells at later stages pos-haploHSCT.

**Table 5:** Bivariate correlation analysis between recoveries of T cell subpopulations and EBV reactivation after 10 mg/kg ATG-conditioned haploHSCT

		Day 30					Day 60					Day 90		Day 180	
EBV	T-cell subsets	CD4^+^	CD4^+^CD45RA^+^	CD4^+^CD45RO^+^	CD8^+^CD28^+^	CD4^−^CD8^−^	CD4^+^	CD4^+^CD45RA^+^	CD4^+^CD45RO^+^	CD8^+^CD28^+^	CD4^−^CD8^−^	CD8^+^CD28^+^	CD4^−^CD8^−^	CD8^+^CD28^+^	CD4^−^CD8^−^
	Correlation coefficient	−0.216	−0.242	−0.212	−0.208	−0.378	0.088	0.063	0.021	0.130	−0.122	−0.020	−0.352	0.105	−0.021
	Sig. (2-tailed)	0.243	0.189	0.252	0.261	0.036	0.643	0.741	0.912	0.493	0.521	0.921	0.066	0.580	0.912

Next, we performed a correlation analysis between the recoveries of T cell subpopulations and the incidence of EBV reactivation for all subjects, irrespective of the ATG dose. As presented in Table 6, the recoveries of CD4^−^CD8^−^ T cells on day 30 (Spearman’s r_p_ = -0.313, *P* = 0.015) and day 90 (Spearman’s r_p_ = −0.318, *P* = 0.019) were significantly correlated to the occurrence of EBV reactivation. Taken together, our data suggested that CD4^−^CD8^−^ T cells could be an important subpopulation that plays a role in response to EBV infection after haploHSCT.

**Table 6:** Bivariate correlation analysis between recoveries of T cell subpopulations and EBV reactivation after haploHSCT

		Day 30					Day 60					Day 90		Day 180	
EBV	T−cell subsets	CD4^+^	CD4^+^CD45RA^+^	CD4^+^CD45RO^+^	CD8^+^CD28^+^	CD4^−^CD8^−^	CD4^+^	CD4^+^CD45RA^+^	CD4^+^CD45RO^+^	CD8^+^CD28^+^	CD4^−^CD8^−^	CD8^+^CD28^+^	CD4^−^CD8^−^	CD8^+^CD28^+^	CD4^−^CD8^−^
	Correlation coefficient	−0.214	−0.213	−0.300	−0.224	−0.313	−0.035	−0.074	−0.071	−0.011	−0.212	−0.104	−0.318	0.008	−0.142
	Sig. (2-tailed)	0.100	0.102	0.020	0.086	0.015	0.800	0.589	0.605	0.939	0.117	0.456	0.019	0.954	0.306

## Discussion

T-cell depletion with ATG is a common strategy for preventing GVHD in allogeneic HSCT settings. However, the potentially negative impact of ATG has been an issue of debate [16–18]. In the setting of haploHSCT, few studies have demonstrated the influence of ATG dosage modulation on lymphocyte recovery and its association with the risk of viral infections. Consistent with our previous work [15], we showed that high-dose ATG was associated with delayed recoveries of CD19^+^ B cells, CD3^+^ T cells, and CD4^+^ T cells during the first month after haploHSCT. Here, we also showed that high-dose ATG delayed the recoveries of CD4^+^, CD4^+^CD45RA^+^, and CD4^+^CD45RO^+^ T cells for 2 months, delayed the recovery of CD4^−^CD8^−^ T cells for 6 months, and delayed the recovery of CD8^+^CD28^+^ T cells for 12 months after transplantation. Moreover, our bivariate correlation analysis indicated that the persistent delay in CD4^−^CD8^−^ T cell recovery was closely related to an increased risk of EBV infection post-haploHSCT.

Double negative, CD4^−^CD8^−^ T cells are defined by exclusion; they comprise the group of CD3^+^ T cells that do not express CD4 or CD8, and they are infrequently observed in peripheral blood and lymphoid organs [19]. Traditionally, studies on double-negative T cells have predominantly focused on the negative effects involved in autoimmune syndromes and inflammation [20, 21]. A recent review proposed potential roles of double-negative T cells in the normal immune system [22]. Although functional analyses of double-negative T cells lagged behind analyses of well-known T cell subpopulations, normal double-negative T cells have emerged as potent effector cells involved in host defense [23]. A previous study demonstrated that double-negative T cells comprised a major host-response T cell subset in the lungs of mice during inhalational Francisella infections [24]. In the context of HSCT, the characteristics of double-negative T-cell recovery have not been documented. The present study was the first to report that high-dose ATG conditioning significantly delayed the recovery of CD4^−^CD8^−^ T cells after haploHSCT, and this delay was correlated with an increased risk of EBV reactivation. These novel findings suggested that CD4^−^CD8^−^ T cells are important in protecting against viral infections; however, we could not rule out the contributions of other functional T cell subpopulations. In addition, CD4^−^CD8^−^ T cells comprise a mixed subset which includes cells that express either αβ or γδ T cell receptors and, other cells that are incompletely characterized [19]. It would be interesting to identify which subset of CD4^−^CD8^−^ T cells functions in protecting against EBV reactivation after HSCT.

Apart from EBV and CMV, other viruses, like adenovirus (AdV), BK virus (BKV), and human herpes virus 6 (HHV6), can potentially cause severe infections after allogeneic HSCT [25–27]. These three viruses were recently added into the list of viruses that require monitoring in routine examinations for recipients of haploHSCT in our institute. The question of whether the incidences of AdV, BKV, and HHV6 reactivation are affected by different conditioning doses of ATG awaits future investigations.

We previously reported that administration of 10 mg/kg ATG resulted in a significant decrease in the occurrence of severe aGVHD compared to a 6 mg/kg ATG dose [15]. In the current study, the incidences of grades III-IV aGVHD were not statistically different between the groups, although the ratios of incidences in the ATG-10 versus ATG-6 group were 6.5 % (2/31) and 13.8 % (4/29; Table 4). The failure to observe a difference could be attributed to the limited size of the present retrospective study.

Both the dosage and timing of ATG administration have been suggested as critical factors in T-lymphocyte recovery after HSCT [9]. The present study showed that 6 mg/kg ATG was related to a faster recovery of T cell subsets and lower incidence of EBV infection after haploHSCT, compared to 10 mg/kg AGT. Our previous study showed a significant reduction in the occurrence of severe aGVHD with 10 mg/kg ATG treatment. Because the overall survival rates were comparable between the ATG-6 and ATG-10 groups [15], we currently use 10 mg/kg ATG to reduce the occurrence of aGVHD. Further investigation is required to determine whether an intermediate ATG dose might preserve the protective effect of preventing GVHD without delaying T-cell recovery. We recently initiated a multi-center clinical trial to compare the outcomes of recipients of haploHSCT that received 10 versus 7.5 mg/kg ATG. We hope the results of that trial will facilitate optimization of the ATG conditioning dose. On the other hand, it has been shown that conditioning with ATG closer to the day of transplantation and longer active ATG exposure were correlated with slower T-cell recovery after HSCT [28, 29]. In the present study, blood concentrations of active ATG after haploHSCT were not monitored. However, evaluation of the time of lymphocyte exposure to ATG and its impact on T-cell reconstitution may also facilitate optimization of the conditioning dosage and timing pre-haploHSCT.

## Conclusions

The present study demonstrated that 10 mg/kg ATG, compared to 6 mg/kg ATG, significantly hampered the recoveries of some T-cell subpopulations in a time-dependent manner, post-haploHSCT. Our study was the first to connect the recovery of CD4^−^CD8^−^ T cells to the risk of EBV infection after HSCT. Future prospective and large cohort studies should provide more unbiased evidences to clarify our findings and benefit the overall outcome in recipients of haploHSCT.
